# O01 Epidemiology and clinical characteristics of patients with carbapenem-resistant Enterobacterales infections: experience from a large tertiary care centre in a developing country

**DOI:** 10.1093/jacamr/dlad143.001

**Published:** 2024-01-03

**Authors:** Banan M Aiesh, Yazan Maali, Farah Qandeel, Siwar Omarya, Shatha Abu Taha, Suha Sholi, Ali Sabateen, Adham Abu Taha, Sa’ed H Zyoud

**Affiliations:** Department of Infection Prevention and Control, An-Najah National University Hospital, Nablus, 44839, Palestine; Department of Medicine, College of Medicine and Health Sciences, An-Najah National University, Nablus 44839, Palestine; Department of Medicine, College of Medicine and Health Sciences, An-Najah National University, Nablus 44839, Palestine; Department of Medicine, College of Medicine and Health Sciences, An-Najah National University, Nablus 44839, Palestine; Department of Medicine, College of Medicine and Health Sciences, An-Najah National University, Nablus 44839, Palestine; Department of General Surgery, An-Najah National University Hospital, Nablus, 44839, Palestine; Department of Infection Prevention and Control, An-Najah National University Hospital, Nablus, 44839, Palestine; Department of Pathology, An-Najah National University Hospital, Nablus, 44839, Palestine; Clinical Research Center, An-Najah National University Hospital, Nablus, 44839, Palestine

## Abstract

**Background:**

Carbapenem-resistant (CR) Enterobacterales (CREs) are a significant source of healthcare-associated infections. These bacteria are difficult to treat and have a high mortality rate due to high rates of antibiotic resistance. These pathogens are also linked to major outbreaks in healthcare institutions, especially those with limited resources in infection prevention and control.

**Objectives:**

To describe the epidemiology and clinical characteristics of patients with carbapenem-resistant Enterobacteriaceae in a referral hospital in a developing country.

**Methods:**

This was a retrospective cross-sectional study that included 218 patients admitted to An-Najah National University Hospital between 1 January 2021, and 31 May 2022. The target population was all patients with CRE infection or colonization in the hospital setting.

**Results:**

Of the 218 patients, 135 had CR *Klebsiella pneumoniae* (61.9%) and 83 had CR *Escherichia coli* (38.1%). Of these, 135 were male (61.9%) and 83 were female (38.1%), with a median age of 51 years (IQR 24–64). Malignancy was a common comorbidity in 36.7% of the patients. Approximately 18.3% of CRE patients were obtained from patients upon admission to the emergency department, the largest percentage among departments. Most CRE pathogens were isolated from rectal swabs, accounting for 61.3%. Among the 218 patients, colistin was the most widely used antimicrobial agent (13.3%). CR *E. coli* showed resistance to amikacin in 23.8% of the pathogens tested and 85.7% for trimethoprim/sulfamethoxazole compared with CR *K. pneumoniae*, for which the resistance to trimethoprim/sulfamethoxazole was 74.1%, while for amikacin it was 64.2%. Regarding meropenem MIC, 85.7% of CR *E. coli* were greater than 16 mg/L compared with 84% of CR *K. pneumoniae* isolates.

**Conclusions:**

This study found that CREs are frequently reported in this tertiary care setting, implying the presence of selective pressure and transmission associated with healthcare setting. The antibiotics tested showed a variety of resistance rates, with CR *K. pneumoniae* being more prevalent than CR *E. coli* and exhibiting an extremely high resistance pattern to the available therapeutic options.

